# Optimizing Autonomous Vehicle Performance Using Improved Proximal Policy Optimization

**DOI:** 10.3390/s25061941

**Published:** 2025-03-20

**Authors:** Mehmet Bilban, Onur İnan

**Affiliations:** 1 Computer Technologies, Necmettin Erbakan University, 42360 Seydişehir, Turkey; mbilban@erbakan.edu.tr; 2 Computer Engineering, Faculty of Technology, Selçuk University, 42140 Konya, Turkey

**Keywords:** autonomous vehicles, apache kafka, CARLA simulator, levy flight, proximal policy optimization

## Abstract

**Highlights:**

In this study, a new Lévy flight-integrated proximal policy optimization (LFPPO) algorithm for enhanced exploration and control in autonomous vehicles is introduced. This enables autonomous vehicles to overcome the exploration limitations of standard PPO algorithms, providing improved decision-making and enhanced control over speed and acceleration, especially in complex urban environments. Indeed, the LFPPO algorithm achieves superior performance and reliability in dynamic driving scenarios: the experimental results in the CARLA simulator show that the LFPPO algorithm significantly outperforms the standard PPO algorithm, achieving a success rate of 99% (vs. 81%), and exhibiting a robust and reliable autonomous driving performance with optimized speed and acceleration control in dynamic urban traffic scenarios.

**What are the main findings?**

- The integration of Lévy flight into the proximal policy optimization (PPO) algorithm (LFPPO) significantly improves the algorithm’s exploration capabilities, allowing it to escape local minima and achieve better policy optimization.
- The experimental results in the CARLA simulator show that the LFPPO algorithm achieves a 99% success rate, compared to the 81% achieved by the standard PPO algorithm, demonstrating enhanced stability and higher rewards in autonomous vehicle decision-making.

**What is the implication of the main finding?**

- The LFPPO algorithm enables autonomous vehicles to make more reliable and safer decisions in complex and dynamic traffic conditions, enhancing overall driving performance.
- The integration of real-time data streaming using Apache Kafka allows autonomous systems to process and react to dynamic environments more efficiently, improving real-time decision-making.

**Abstract:**

Autonomous vehicles must make quick and accurate decisions to operate efficiently in complex and dynamic urban traffic environments, necessitating a reliable and stable learning mechanism. The proximal policy optimization (PPO) algorithm stands out among reinforcement learning (RL) methods for its consistent learning process, ensuring stable decisions under varying conditions while avoiding abrupt deviations during execution. However, the PPO algorithm often becomes trapped in a limited search space during policy updates, restricting its adaptability to environmental changes and alternative strategy exploration. To overcome this limitation, we integrated Lévy flight’s chaotic and comprehensive exploration capabilities into the PPO algorithm. Our method helped the algorithm explore larger solution spaces and reduce the risk of getting stuck in local minima. In this study, we collected real-time data such as speed, acceleration, traffic sign positions, vehicle locations, traffic light statuses, and distances to surrounding objects from the CARLA simulator, processed via Apache Kafka. These data were analyzed by both the standard PPO and our novel Lévy flight-enhanced PPO (LFPPO) algorithm. While the PPO algorithm offers consistency, its limited exploration hampers adaptability. The LFPPO algorithm overcomes this by combining Lévy flight’s chaotic exploration with Apache Kafka’s real-time data streaming, an advancement absent in state-of-the-art methods. Tested in CARLA, the LFPPO algorithm achieved a 99% success rate compared to the PPO algorithm’s 81%, demonstrating superior stability and rewards. These innovations enhance safety and RL exploration, with the LFPPO algorithm reducing collisions to 1% versus the PPO algorithm’s 19%, advancing autonomous driving beyond existing techniques.

## 1. Introduction

Autonomous vehicles (AVs) represent a transformative innovation in modern engineering and artificial intelligence, defined as vehicles capable of independent movement and decision-making without human intervention. These vehicles depend on advanced data collection, processing, and analysis systems to ensure safe operation in complex and dynamic traffic conditions. Core AV technology integrates data from radar, LiDAR, cameras, and ultrasonic sensors, processed by high-performance computing, to enable three-dimensional environmental perception [1].

The World Health Organization reports that 90% of traffic accidents result from human error [2]. Equipped with precise sensors and accurate algorithms, AVs can mitigate these errors, reducing accident rates. Furthermore, through vehicle-to-vehicle (V2V) and vehicle-to-infrastructure (V2I) communication, AVs optimize traffic flow, enhancing efficiency in lane changes and speed adjustments [3,4]. Precise driving and optimization algorithms also lower fuel consumption and carbon emissions, significantly contributing to environmental sustainability [3]. However, AV deployment encounters substantial challenges, including regulatory inconsistencies across regions, ethical dilemmas in unavoidable crash scenarios, and public acceptance barriers due to safety concerns and trust deficits [4,5,6]. These challenges, including sensor misperceptions in adverse weather, rapid data processing demands, and complex ethical decision-making, underscore the need for advanced algorithms such as the LFPPO algorithm, which enhances adaptability and safety to address these deployment barriers and ensure safe, efficient operation in dynamic urban environments [5,6,7]. Overcoming these obstacles requires progress in sensor technology, data processing, and machine learning algorithms for robust decision-making in unpredictable traffic scenarios.

Such challenges, stemming from sensor limitations, data processing difficulties, and ethical decision-making needs, significantly impact the performance of autonomous vehicles in urban environments. Overcoming these obstacles requires advancements in sensor technologies, data processing algorithms, and the machine learning algorithms used in decision-making processes to ensure the safe and efficient operation of autonomous vehicles in complex and unpredictable traffic scenarios.

Machine learning methods are frequently used to address challenges in various fields, such as disease diagnosis and signal classification [8,9,10,11,12,13,14], as well as detecting fake content and processing textual data [15,16]. Similarly, machine learning plays a critical role in overcoming the challenges faced by autonomous vehicles. Deep learning algorithms process data from sensors and cameras to improve object detection and classification, enabling vehicles to more accurately perceive pedestrians, other vehicles, and obstacles [17,18,19,20,21,22,23,24].

Reinforcement learning (RL) algorithms help autonomous vehicles analyze different driving scenarios and choose the most appropriate actions. Algorithms like PPO enable vehicles to perform safely and stably under varying driving conditions [25,26,27]. In particular, integrating the PPO algorithm with chaotic systems and active learning frameworks significantly improves vehicle optimization and control processes [28]. These advancements allow vehicles to explore broader search spaces and utilize high-quality, diverse training datasets, thereby enabling them to handle challenging situations more effectively. Moreover, machine learning methods accelerate the learning process and enhance vehicle performance by training them on larger datasets. The PPO algorithm, in particular, is effective in optimization processes and controlling chaotic systems [28], and its ability to explore wider search spaces contributes to achieving better outcomes [29]. The continuous development and application of such methods offer tremendous potential to enhance the capabilities of autonomous and connected vehicles in complex environments.

The CARLA simulator provides researchers with a valuable tool for developing autonomous vehicles capable of handling complex real-world tasks. CARLA offers realistic environments for testing various driving scenarios, such as urban traffic, highway driving, and obstacle avoidance [1]. Additionally, it supports server–client architecture, enabling the testing of diverse algorithms under varying weather conditions, traffic densities, and intersection scenarios. The features provided by CARLA significantly reduce the costs and risks associated with real-world testing. Its open-source structure fosters collaboration among researchers and accelerates the advancement of autonomous driving technologies [30].

Autonomous vehicles operate in complex and ever-changing environments such as urban traffic. Under these dynamic conditions, they require a reliable and stable learning mechanism to make accurate and quick decisions. The PPO algorithm provides a more stable learning process compared to other RL algorithms [31,32,33]. Its stability ensures that autonomous vehicles can make consistent decisions under changing conditions and avoid abrupt deviations while implementing them. This stability is crucial in scenarios involving sudden changes, such as pedestrian movements, maneuvers by other vehicles, and unexpected environmental factors.

Autonomous vehicles continuously learn from data streams, and they must use these data efficiently. The PPO algorithm increases data efficiency by allowing the reuse of collected experiences. This enables vehicles to learn about their surroundings more quickly and adopt appropriate behaviors more rapidly. For example, the PPO algorithm’s efficient use of data allows vehicles to learn frequent scenarios faster and respond to them more effectively. This improves performance in common driving scenarios such as recognizing traffic signs, stop-and-go movements, or sudden accelerations/decelerations [31,32,33].

For autonomous vehicles to adapt to constantly changing environmental factors, they must explore a wide range of policy spaces. The PPO algorithm enables the stable exploration of these policy spaces by allowing policies to be updated within a defined range while maintaining learning stability. This capability helps vehicles better perceive their environment and develop appropriate behaviors based on that perception. For instance, in a crowded intersection, the PPO algorithm can help assess environmental factors and select the safest and fastest crossing strategy [31,34].

Although the PPO algorithm generally provides a stable learning process, unstable updates can occur under certain conditions. The chaotic step mechanism of Lévy flight mitigates these instabilities, making the learning process more robust. This mechanism helps prevent sudden performance drops and fluctuations during training.

Existing studies have implemented limited strategies to enhance the exploration capabilities of the PPO algorithm. This study addresses this gap by integrating the Lévy flight strategy with the PPO algorithm. The primary aim is to combine Lévy flight with the PPO algorithm to improve decision-making processes for autonomous vehicles under complex and dynamic traffic conditions. The chaotic and extensive search capability of Lévy flight significantly enhances the exploration capacity of the PPO algorithm and reduces the likelihood of getting stuck in local minima.

In the literature, there is no comprehensive study that examines the integration of the LFPPO algorithm in detail while also utilizing Apache Kafka for real-time data processing. The findings of this study indicate that the LFPPO algorithm achieved a 99% success rate, compared to the standard PPO algorithm, which was limited to 81%. Additionally, the LFPPO algorithm demonstrated a more stable and reliable performance.

The PPO algorithm is widely recognized in the literature for its stable and reliable learning process. However, its limited exploration capacity often causes the algorithm to get stuck in local minima and fail to achieve optimal results in complex conditions. This study integrates the Lévy flight strategy into the PPO algorithm to expand its exploration capabilities and achieve more effective driving performance. By utilizing the chaotic and extensive search structure of Lévy flight, the algorithm’s exploration capacity is broadened, enabling it to overcome local minimum problems. Furthermore, this study tests this strategy in an autonomous vehicle simulation using Apache Kafka as a real-time data processing platform. The results demonstrate that the LFPPO algorithm outperformed the traditional PPO algorithm in terms of rewards and success rates. While the PPO algorithm’s stability is well-documented, its constrained exploration often limits its effectiveness in unpredictable environments. The LFPPO algorithm addresses this by integrating Lévy flight’s chaotic step mechanism, enabling broader policy exploration and reducing convergence to local optima a challenge unaddressed by existing PPO variants like MA-PPO [33] or CPPO [35]. Furthermore, the incorporation of Apache Kafka for real-time data processing distinguishes the LFPPO algorithm from prior RL methods, offering a scalable framework for dynamic decision-making. These novel contributions not only enhance autonomous vehicle performance but also provide new insights into RL exploration strategies, advancing the state-of-the-art in complex, real-world applications.

To elucidate these contributions, this paper is structured as follows: Section 2 reviews related work, highlighting existing RL approaches and their limitations in autonomous driving. Section 3 details the materials and methods, including the mathematical foundations of the PPO and LFPPO algorithms, and Apache Kafka. Section 4 presents the evaluation criteria and mathematical representations used to assess performance. Section 5 describes Apache Kafka’s core components and operational principles. Section 6 outlines the hyperparameter tuning process and selected values for the PPO and LFPPO algorithms. Section 7 reports experimental results, including safety and comfort metrics from CARLA simulations. Finally, Section 8 offers conclusions and recommendations for future research, emphasizing the LFPPO algorithm’s practical implications and potential advancements.

## 2. Related Works

### 2.1. Literature Review

Chen et al. proposed an adaptive federated reinforcement learning (FRL) framework integrating the proximal policy optimization (PPO) algorithm with federated learning (FL) to enhance decentralized learning for autonomous driving. This framework employed the adaptive federated optimization strategies FedYogi, FedAvg, FedAdam, and FedAdagrad to accelerate convergence and improve policy generalization. Experiments showed that FedYogi outperformed the traditional PPO algorithm and other FRL approaches, achieving faster convergence and lower variance after 100,000 training steps [36].

Guan et al. proposed a model-accelerated proximal policy optimization (MA-PPO) framework to enhance centralized coordination of autonomous vehicles at intersections without traffic lights. The method integrated an environmental model into the PPO framework to improve sample efficiency and reduce computational costs. The experimental results demonstrated that MA-PPO reduced computation time to 1/400 of that required by model predictive control (MPC) methods while increasing intersection efficiency by 4.5 times. The approach ensured safe and efficient vehicle movement by optimizing decision-making in multi-agent traffic environments through centralized cooperation. This study contributed to the literature by presenting a novel RL-based centralized cooperation framework that significantly improved learning efficiency, reduced reliance on real-world data, and optimized intersection control strategies [33].

Peng et al. proposed a curriculum proximal policy optimization (CPPO) framework with stage-decaying clipping to enhance self-driving decision-making at unsignalized intersections. The CPPO method incorporated curriculum learning into the proximal policy optimization (PPO) algorithm by adjusting the clipping parameter dynamically across training stages, enabling the agent to first explore with a large parameter and later refine its policy with a smaller one. This strategy accelerated training convergence and improved generalization. Comparative experiments in Highway_Env showed that CPPO outperformed the standard PPO algorithm in both training speed and success rate. Specifically, CPPO achieved a 100% success rate in simple intersection scenarios and maintained 78.5% success with four surrounding vehicles, surpassing baseline PPO methods. The study contributed to RL for autonomous driving by introducing a curriculum-based clipping strategy that improved sample efficiency and policy adaptability in highly dynamic environments [35].

Muhammad et al. provided a comprehensive review of deep learning [30] techniques for enhancing the safety of autonomous driving (AD) systems. Their study categorized DL-based methods into three key stages: measurement, analysis, and execution, covering essential tasks such as road detection, lane detection, pedestrian detection, drowsiness detection, and collision avoidance. The paper evaluated various DL architectures, including convolutional neural networks (CNNs), recurrent neural networks (RNNs), and transformer-based models, analyzing their impact on autonomous vehicle perception and decision-making. The review highlighted that state-of-the-art pedestrian detection models achieve an average accuracy of 85% in ideal conditions, but performance drops to 65% in complex urban scenarios. The authors also discussed key challenges, such as data bias, real-time processing limitations, and the lack of interpretability in deep learning models for AD. This work contributed to the literature by providing a roadmap for improving safety-centric DL models, identifying critical gaps, and suggesting future research directions for enhancing the robustness and reliability of DL-based autonomous driving systems [17].

Yuan et al. integrated deep RL (DRL) with game theory (GT) to enhance decision-making for autonomous vehicles at unsignalized intersections. Their framework modeled vehicles as strategic agents with diverse driving behaviors (conservative, aggressive, adaptive), using cognitive hierarchy reasoning to improve interaction prediction. Simulated in a ROS-Gazebo environment, the approach showed a high alignment between simulated and real-world performance, improving intersection safety without explicit vehicle-to-vehicle communication. This study contributed to the field by providing a scalable multi-agent RL model that reduced dependency on costly real-world testing [26].

Yu et al. introduced an occlusion-aware risk assessment framework to improve autonomous driving safety in urban environments with limited sensor range. By utilizing road layout data and predicting occluded risks, the proposed approach enabled proactive driving behavior adjustments. Their experimental results indicated that this method achieved a 4.8× reduction in collision rates compared to baseline models while improving ride comfort. The study contributed to risk-aware trajectory planning, ensuring safer decision-making in occlusion-heavy urban driving scenarios [7].

Xu et al. developed a multi-objective approximate policy iteration (MO-API) algorithm to optimize lane-changing and overtaking decisions in highway environments. Their method integrated value function approximation and feature learning, leading to enhanced decision efficiency. Experimental evaluations in a 14-degree-of-freedom highway simulation demonstrated that the approach outperformed traditional multi-objective Q-learning in terms of learning efficiency. This study advanced RL-based highway driving strategies, providing a data-driven approach for adaptive lane-changing policies [25].

Grandesso et al. presented continuous actor-critic trajectory optimization (CACTO), a RL framework combining trajectory optimization (TO) with RL to improve continuous control in nonlinear dynamical systems. The proposed method enhanced policy learning by avoiding local minima and improving control stability. Experimental results in vehicle control applications showed that CACTO surpassed the deep deterministic policy gradient (DDPG) and PPO algorithms in efficiency and stability. This study contributed to policy search efficiency and optimal trajectory planning, making it well-suited for real-world autonomous driving applications. problems [37].

Yau et al. applied the PPO algorithm to nonlinear chaotic systems, such as the Lorenz chaotic system, without requiring prior system equations. Their study demonstrated that the PPO algorithm-based controller effectively suppressed chaos while improving stability under different initial conditions. Compared to traditional controllers, the PPO approach exhibited faster response times and better adaptability. This research highlighted the potential of deep RL in nonlinear system stabilization, extending beyond autonomous driving into complex control systems [28].

Rievaj et al. analyzed the potential benefits of autonomous vehicles (AVs) in reducing traffic congestion, emissions, and road accidents. Their study highlighted that automation can significantly lower road fatalities, improve fuel efficiency, and optimize urban mobility. The research suggested that adaptive cruise control and self-driving capabilities could enhance road capacity while minimizing environmental impact. This study provided a comprehensive assessment of the long-term economic and environmental implications of autonomous vehicles [3].

Ferrarotti et al. examined the interaction between autonomous and human-driven vehicles in roundabouts, using SUMO traffic simulation and RL-based driving policies. Their findings indicated that with 80% AV presence, crossing times improved by 10.72%, and pollution levels decreased by 38.98%. The study demonstrated that higher AV penetration enhanced traffic flow and safety, offering insights into urban planning for autonomous mobility [34].

Martínez-Díaz et al. reviewed the theoretical and practical challenges of deploying autonomous vehicles, focusing on sensor limitations, V2V cooperation, and regulatory barriers. Their analysis identified that real-time obstacle detection and liability issues remain significant hurdles in large-scale AV adoption. This study provided a roadmap for future research directions, emphasizing the need for improved perception systems and legal frameworks to support autonomous driving [5].

Huang et al. proposed the subsection-proximal policy optimization (subsection-PPO) algorithm, an improved PPO-based RL approach for adaptive vehicle-following control. The method optimized acceleration and braking strategies, reducing collision risks in dense traffic environments. Experimental results confirmed that Subsection-PPO enhanced safety and efficiency compared to conventional vehicle-following controllers. This study contributed to deep RL-based adaptive control strategies, optimizing AV behavior in mixed-traffic conditions [32].

Nastjuk et al. investigated the factors influencing the acceptance of autonomous driving from an end-user perspective through a mixed-methods approach that combined qualitative research with a large-scale online survey. By applying the technology acceptance model (TAM), they analyzed 316 participants’ responses to determine the key factors affecting the consumer adoption of autonomous vehicles. Their findings revealed that social influence, system characteristics, and personal attitudes positively impacted user acceptance, while concerns related to cost, privacy, and safety acted as barriers to adoption. This study provided valuable insights for policymakers and automotive manufacturers to address public concerns and improve adoption rates [4].

Galvao et al. presented a comprehensive review of pedestrian and vehicle detection techniques used in autonomous vehicle (AV) perception systems. The study compared traditional computer vision methods and deep learning-based approaches, concluding that deep learning consistently outperformed classical techniques in terms of detection accuracy and robustness. However, the authors highlighted challenges in low-light and occluded scenarios, where detection accuracy declined. The study identified a critical need for diverse and high-quality datasets to improve real-world performance and proposed future research directions focused on enhanced occlusion-handling models for AV perception systems [18].

Ashraf et al. proposed an optimized deep deterministic policy gradient (DDPG) framework for autonomous driving by tuning its hyperparameters using the whale optimization algorithm (WOA). The approach optimized key parameters, including learning rates, discount factor, target network update rate, and batch size, to improve training efficiency and learning stability. The experiments, conducted in TORCS (the open racing car simulator), demonstrated that the optimized DDPG model outperforms baseline hyperparameter settings, resulting in better driving stability and higher cumulative rewards. This study contributed to deep RL in autonomous driving by introducing an effective hyperparameter optimization technique that enhanced real-time performance and driving efficiency [38].

The proximal policy optimization (PPO) algorithm has emerged as a robust RL algorithm due to its stability and efficiency, as demonstrated by Schulman et al. [39]. However, its reliance on a clipped surrogate objective limits its exploration capacity, often causing convergence to suboptimal policies in complex environments. Recent variants, such as Curriculum PPO (CPPO) by Peng et al. [35], introduced stage-decaying clipping to enhance training convergence, achieving a 78.5% success rate in unsignalized intersections. Similarly, Guan et al.’s model-accelerated PPO (MA-PPO) algorithm [33] improved sample efficiency for intersection coordination but lacked mechanisms for robust exploration in unpredictable urban settings. These studies highlight a critical gap in PPO-based methods: the inability to effectively explore diverse policy spaces, which the LFPPO algorithm addresses through Lévy flight integration.

Beyond reinforcement learning approaches like the PPO algorithm, meta-heuristic algorithms inspired by natural phenomena have gained traction in autonomous systems, particularly for path planning tasks. For instance, Chen et al. [40] proposed the multi-strategy improved gray wolf optimization (MSIAR-GWO) algorithm, enhancing the traditional GWO by integrating reinforcement learning for parameter tuning, Lévy flight, and Brownian motion for improved exploration, and a dynamic reverse learning strategy to maintain population diversity. Tested in the context of mobile robot path planning, MSIAR-GWO demonstrated superior convergence speed and solution accuracy compared to classical GWO and other swarm intelligence methods, achieving shorter and smoother paths in complex grid-based environments. This work parallels the use of Lévy flight in LFPPO to escape local optima, highlighting a broader trend of incorporating chaotic exploration strategies into optimization frameworks for autonomous navigation. Such approaches complement RL-based methods by offering robust alternatives for scenarios where real-time adaptability and computational efficiency are critical.

In addition to RL-based methods, meta-heuristic algorithms have been increasingly explored for optimizing sensor-driven systems, particularly in wireless sensor networks (WSNs). Zheng et al. [41] introduced an enhanced flower pollination algorithm with gaussian perturbation (EFPA-G), which improved the traditional FPA by integrating Lévy flight for global exploration and Gaussian perturbation for enhanced local exploitation. Applied to the DV-Hop method for WSN node localization, EFPA-G achieved superior accuracy (NRMSE of 19.52% compared to 20.27% for state-of-the-art methods) by leveraging sensor connectivity data, demonstrating its effectiveness in real-world sensor network applications. This approach shares similarities with the LFPPO algorithm’s use of Lévy flight to escape local optima in autonomous vehicle decision-making, underscoring a broader trend of combining chaotic exploration strategies with optimization frameworks. By addressing the balance between exploration and exploitation, EFPA-G complements RL-based approaches like the LFPPO algorithm, particularly in contexts where sensor data processing is critical, aligning with the growing emphasis on robust, sensor-centric solutions in autonomous systems.

Lévy flight, a random walk strategy characterized by a mix of short steps and infrequent long jumps, has been widely applied to enhance exploration in optimization problems [40]. For instance, Chen et al. [40] utilized Lévy flight in a grey wolf optimization framework for mobile robot path planning, demonstrating its ability to escape local optima. In RL, Yau et al. [28] explored the PPO algorithm with chaotic systems but did not leverage Lévy flight’s unique step distribution for broader exploration. The integration of Lévy flight into the PPO algorithm, as proposed in this study, represents a novel application to autonomous driving, enabling the algorithm to navigate complex urban scenarios more effectively than traditional methods.

Real-time data processing is critical for autonomous vehicles operating in dynamic environments. While most RL studies rely on static datasets or simulated batches, few incorporate distributed streaming platforms like Apache Kafka. Nastjuk et al. [4] emphasized the importance of real-time sensor data for user acceptance, but their work lacks an RL integration. In contrast, this study leverages Kafka to stream CARLA simulator data, enabling LFPPO to process dynamic inputs efficiently—a feature distinguishing it from prior PPO-based approaches that lack real-time adaptability.

Beyond the PPO algorithm, other RL algorithms like the deep deterministic policy gradient (DDPG) and soft actor-critic algorithms have been applied to autonomous driving. Ashraf et al. [38] optimized DDPG hyperparameters using the whale optimization algorithm, improving stability in TORCS simulations. However, DDPG struggles with high-dimensional action spaces, a challenge the PPO algorithm mitigates through its policy gradient approach. SAC, known for its entropy maximization, enhances exploration but at the cost of computational complexity [42]. The LFPPO algorithm builds on the PPO algorithm’s stability while introducing Lévy flight for efficient exploration, offering a balanced alternative to these methods in complex urban traffic scenarios.

In the literature, there is no comprehensive study that examines the integration of the LFPPO algorithm in detail while also utilizing Apache Kafka for real-time data processing. The findings of this study indicate that the LFPPO algorithm achieved a 99% success rate, compared to the standard PPO algorithm, which was limited to 81%. Additionally, the LFPPO algorithm demonstrated more stable and reliable performance. The PPO algorithm is widely recognized in the literature for its stable and reliable learning process. However, its limited exploration capacity often causes the algorithm to get stuck in local minima and fail to achieve optimal results in complex conditions. This study integrates the Lévy flight strategy into the PPO algorithm to expand its exploration capabilities and achieve more effective driving performance. By utilizing the chaotic and extensive search structure of Lévy flight, the algorithm’s exploration capacity is broadened, enabling it to overcome local minimum problems. Furthermore, this study tests this strategy in an autonomous vehicle simulation using Apache Kafka as a real-time data processing platform. The results demonstrate that the LFPPO algorithm outperformed the traditional PPO in terms of rewards and success rates.

While the literature demonstrates significant progress in RL for autonomous driving, key gaps remain in exploration efficiency, adaptability to dynamic conditions, and real-time data integration. PPO-based methods excel in stability but falter in exploring diverse policy spaces, as seen in [33,35]. Alternative RL approaches like DDPG and SAC address exploration to some extent but introduce complexity or instability [38,42]. Moreover, the absence of real-time data streaming in most studies limits their practical deployment. This study bridges these gaps by introducing the LFPPO algorithm, which combines the PPO algorithm’s stability with Lévy flight’s chaotic exploration and Apache Kafka’s real-time processing, offering a novel and comprehensive solution for autonomous vehicle decision-making in complex urban environments.

### 2.2. Research Gap and Novel Contributions of This Study

RL approaches for autonomous driving, including the proximal policy optimization (PPO) algorithm [39], face limitations in exploration efficiency, adaptability, and real-time data processing. The PPO algorithm’s clipped objective restricts policy exploration, often yielding suboptimal results in complex urban settings [33,35], a challenge shared by variants like CPPO [35] and MA-PPO [33], as well as methods like DDPG [38]. Most RL studies rely on static datasets, neglecting the dynamic nature of real-world driving and lacking real-time sensor integration [4,17].

This study introduces the LFPPO algorithm, integrating Lévy flight’s chaotic exploration [40] with the PPO algorithm and Apache Kafka’s real-time data streaming to address these gaps. Key contributions include:

1. LFPPO Development: LFPPO enhances exploration by embedding Lévy flight into the PPO algorithm reducing local minima entrapment beyond the standard PPO algorithm and variants [33,35].
2. Real-Time Processing: Using Kafka, LFPPO processes CARLA sensor data (speed, traffic signs) at 20 Hz, enabling dynamic adaptability, absent in prior methods [4,43,44].
3. Robust Validation: Evaluated in CARLA’s Town 10 and Town 5 over 100 episodes and 10 runs, LFPPO achieves a 99% success rate versus the PPO algorithm’s 81%, with safety metrics (1% collision rate) confirming its efficacy.
4. Scalability: the LFPPO algorithm’s architecture supports real-time decision-making, aligning with sensor-centric focus and modern AV trends [17,45].

Supported by a systematic grid search for hyperparameters (*levy_lambda*), the LFPPO algorithm advances RL for autonomous vehicles, balancing increased training time (3242.54 vs. 726.76 s) with superior performance in dynamic environments.

## 3. Materials and Methods

**Figure 1:**
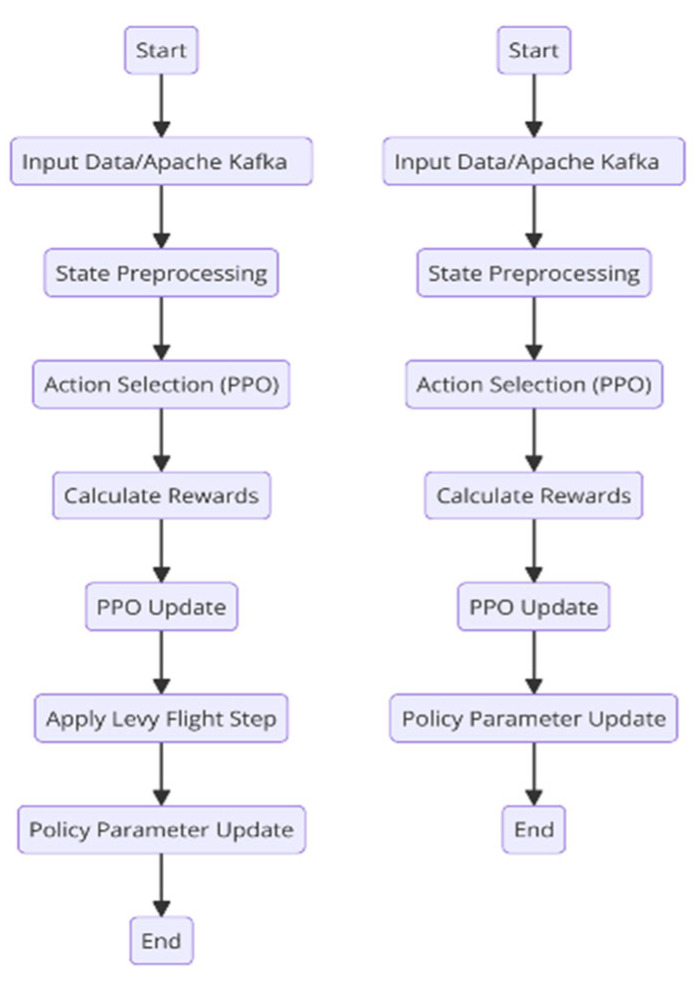
Flowchart illustrating the operational principles of the PPO and LFPPO algorithms, depicting the sequential steps of data collection, advantage computation, and policy updates with Lévy flight integration.

This section mathematically addresses the working principles of the PPO algorithm, LFPPO algorithm, and Apache Kafka. The fundamental operation of PPO and LFPPO algorithms is illustrated in Figure 1.

### 3.1. PPO Algorithm

RL has proven to be an effective method for autonomous driving models [30,43]. RL algorithms aim to learn optimal control strategies by providing feedback in the form of rewards or penalties [36]. During policy updates in RL algorithms, large changes can destabilize the system and lead to unexpected decisions by the vehicle. PPO is a popular RL algorithm known for its stability and effectiveness in addressing such challenges [1,30,43]. PPO is a policy-based RL algorithm that uses a “clipped objective” loss function to ensure stable learning. This loss function constrains policy updates, preventing excessive changes and ensuring that the vehicle avoids sudden or risky maneuvers [44]. For autonomous vehicles, this mechanism minimizes unexpected or dangerous actions in traffic environments. Especially in dense traffic and complex scenarios, the safe and stable policy updates provided by the PPO algorithm enable the vehicle to operate optimally and reliably [35,45,46,47]. The loss function enables the comparison of the current policy with the old policy and is optimized using the advantage function. The primary policy loss function that the PPO algorithm aims to optimize is as follows:

(1) $$L^{CLIP}\;\left(\theta \right)={\hat{E}}_{t\;}\left[\min\left(r_{t}\left(\theta \right)\cdot \;{\hat{A}}_{t},\;clip\left(r_{t}\left(\theta \right),\;1-\varepsilon ,\;1+\varepsilon \right)\cdot \;{\hat{A}}_{t}\right)\right]$$

(2) $$r_{t}\left(\theta \right)=\frac{\pi_{\theta }(\alpha_{t}|s_{t})}{\pi_{\theta_{old}}(\alpha_{t}|s_{t})}$$

The ratio obtained by comparing the action probabilities of the current policy with those of the old policy.

Known as the advantage function, ${\hat{A}}_{t}$ is calculated as the difference between rewards and the value function:

$${\hat{A}}_{t}=R_{t}-V(s_{t})$$

The clipping parameter ε is used to limit excessive changes during policy updates, and $\pi_{\theta }(\alpha_{t}|s_{t})$ represents the probability of a specific action under the current policy. This value is also used as a policy value. The policy value is calculated by the policy network utilized in the PPO algorithm. This policy network is a neural network that maps the vehicle’s environmental states (states) to actions.

This neural network handles the action selection process and parameter update steps of the PPO algorithm. The network takes the following state information as input:

1. Vehicle speed: speed
2. Vehicle acceleration: acceleration
3. Coordinates of traffic elements: traffic_location_x, traffic_location_y, traffic_location_z
4. Vehicle coordinates: vehicle_location_x, vehicle_location_y, vehicle_location_z
5. Status of traffic lights: traffic_light_state
6. Distances to other actors: distances_to_actors

**Figure 2:**
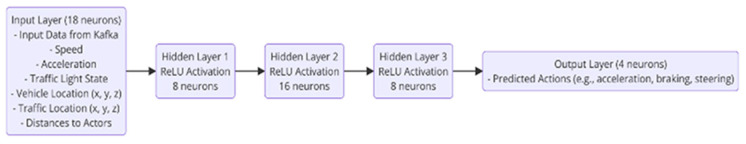
Neural network structure of the PPO algorithm and its action selection processes. This network processes various state information and converts them into actions, enabling the vehicle to respond appropriately to environmental factors.

The neural network processes this state information to compute the probabilities of possible actions (accelerating, speed, braking, steering adjustments). In the output layer, one of these probabilities is selected to determine the vehicle’s response to environmental factors. Figure 2 shows the structure of the neural network in the PPO algorithm and the action selection processes.

### 3.2. Lévy Flight Fundamentals and Use with PPO

Lévy flight is a type of random walk observed frequently in nature, characterized by a combination of long leaps and short movements. This model is particularly useful for optimizing exploration processes in dynamic and uncertain environments. Lévy flight effectively scans wide search areas through long jumps while performing local optimization with shorter steps. Traditional PPO algorithms can sometimes get stuck in local minima during the learning process. This limitation may prevent the algorithm from finding better solutions, forcing it to settle for suboptimal strategies [37]. To address this issue, Lévy flight employs a combination of long and short steps to explore broad search areas and expand the solution space of the algorithm. As a result, the probability of the PPO algorithm getting stuck in local minima decreases, increasing its chances of reaching a global optimum.

The step distribution of Lévy flight is modeled as follows:

(3) $$L\left(s;\;\lambda \right)\sim \;{\left|s\right|}^{-\left(1+\lambda \right)},$$

where *s* represents the step length or distance, determining how large or small the next move will be (as Lévy distribution indicates that large steps occur infrequently but cover great distances, whereas small steps are more common), and λ is a parameter controlling the characteristics of the Lévy flight steps (typically, 0 < λ < 2).

This formula describes how the steps are distributed and how Lévy flight contributes to the exploration strategy; s represents the random step lengths, allowing the Lévy flight algorithm to cover a wide search space.

Lévy flight enhances the parameter update process of the PPO algorithm, as represented by the following equation:

(4) $$\theta_{\left\{t+1\right\}}(L^{LFPPO})=\theta_{t}+\alpha \cdot {𝛻}_{\theta }L^{CLIP\left(\theta \right)}+\beta \cdot L\left(s;\lambda \right),$$

where α represents the learning rate of the PPO algorithm, ${𝛻}_{\theta }L^{CLIP\left(\theta \right)}$ is the gradient of the clipped loss function used in the PPO algorithm, β is the coefficient determining the influence of Lévy flight, and $L\left(s;\;\lambda \right)$ denotes the magnitude of the Lévy flight step, representing a random large or small step and reflecting the chaotic exploration behavior.

The PPO algorithm calculates the advantage function ${\hat{A}}_{t}$ using the reward function value $R_{t}$ as follows:

(5) $${\hat{A}}_{t}=R_{t}-V(s_{t}),$$

Subsequently, the $L^{CLIP\left(\theta \right)}$ loss function is optimized. Lévy flight introduces chaotic and broad exploration steps into policy updates, enhancing the exploration capabilities by updating the θ parameters. This integration allows the algorithm to overcome local minima and improve its exploration efficiency. The pseudocode of the working process of the LFPPO algorithm is shown in Algorithm 1.
**Algorithm** **1:** Working principle of LFPPO algorithm1: Initialize policy network $\pi_{\theta }$ with random weights 2: for each iteration do: 3: Collect experience (s, a, r, s’) using current policy 4: Compute advantage estimates ${Â}_{t}$ = $R_{t}$ − V($s_{t}$) 5: Compute PPO loss function: $\theta_{\left\{t+1\right\}}$ = $\theta_{t}$ + α ∗ ${𝛻}_{\theta }L^{CLIP\left(\theta \right)}$ 6: Apply Lévy flight step: L(s; λ) = $s_{0}$ + $\sum_{i=1}^{n}{\xi_{i}U_{i}}^{-1/\lambda }$ 7: Update policy with combined loss: $\theta_{\left\{t+1\right\}}$ = $\theta_{t}$ + β ∗ L(s; λ) 8: end for

The primary advantage of integrating Lévy flight into the PPO algorithm is its ability to expand the exploration space while maintaining stability in policy updates. Unlike the standard PPO algorithm, which tends to converge to local optima due to restricted step sizes, Lévy flight introduces controlled stochastic jumps that enable broader exploration. This controlled randomness enhances adaptability, particularly in highly dynamic environments where unexpected scenarios frequently occur.

## 4. Evaluation Criteria and Mathematical Representation

In this section, the performance criteria and mathematical foundations used in the evaluation of PPO and LFPPO algorithm are given.

### 4.1. Reward Function

In the PPO algorithm, the reward-penalty function aims to encourage the agent’s goal-oriented behaviors while deterring undesirable actions. The reward function reinforces correct actions of the agent, while the penalty function prevents incorrect or risky behaviors. During the optimization process of the PPO algorithm, rewards and penalties are combined using a mechanism known as the “Clipped Surrogate Objective”. This mechanism limits policy updates, ensuring that the agent learns stably and safely. Consequently, the PPO algorithm effectively supports both the exploration of wide search spaces and the achievement of predefined goals.

(6) $$R_{t}=\sum_{i=1}^{n}\begin{array}{c} C_{i}f_{i}(speed,\;acceleration,\\ \;traffic\_location(x,y,z),\;\\ vehicle\_location(x,y,z),\\ \;traffic\_light\_state,\;distances\_to\_actors) \end{array}$$

where $R_{t}$ is defined as the total reward, $\;C_{i}$ represents the weight coefficient for a specific factor, and $f_{i}(\cdot )$ is a function that calculates the impact of a specific factor on the reward.

### 4.2. Entropy Function

Entropy is used to measure the diversity and exploration rate within a policy. It indicates how uncertain or diverse the model is in its action selections. The mathematical formulation of entropy is as follows:

(7) $$H\left(\pi \right)=-\sum_{\alpha }\pi (\alpha |s)log\;\pi (a|s))$$

where *π*(*α|s*) represents the probability of selecting action α in a given state s.

A high entropy value means the policy is more open to exploration. Low entropy indicates that the policy is more deterministic and engages in less exploration.

### 4.3. Success Rate Function

It measures how long the model remains successful. High success rates improve the model’s consistency and performance in achieving the target. The success rate [2] is defined by the following formula:

(8) $$SR=\frac{N_{success}^{i}}{N_{total}^{i}}$$

where $N_{success}^{i}$ represents the number of successfully completed tasks in the *i*-th episode and $N_{total}^{i}$ represents the total number of tasks in the *i*-th episode.

This metric provides insight into how frequently the vehicle achieves the intended goal relative to the total attempts, demonstrating the reliability and efficiency of the model.

## 5. Apache Kafka’s Core Components and Working Principle

Autonomous vehicles continuously collect large amounts of data from sensors (LIDAR, radar, cameras, etc.) and other onboard systems. Apache Kafka is an ideal platform for processing high-volume data streams and routing these data to other systems in real time. Kafka’s high throughput and low latency capabilities enable efficient processing of vehicle data.

Autonomous vehicles operate within a complex data flow. Kafka’s ability to organize data into different topics simplifies the separation of these data and its distribution to various systems. Additionally, Kafka provides a highly reliable structure for seamless data transmission and storage, ensuring that autonomous vehicles can operate without data loss. Kafka’s distributed architecture allows the system to scale effectively, even under increased load.

Apache Kafka was used to process sensor data such as vehicle locations, traffic light states, and actor distances in real time. Its low latency and high efficiency enabled the fast processing of these data streams and their seamless integration with RL algorithms, including PPO and LFPPO.

The core components of Apache Kafka are:

Producer $\left(P\right)$: Sends data to Kafka.

Kafka Broker (B): Receives data and stores them under specific topics.

Topic (T): A logical partition of the data, each of which can be divided into n partitions ($P_{i})$.

Partition ($P_{i})$: A specific segment of the data, each containing sequential messages ${(m}_{1},m_{2}{,\ldots \ldots ,m}_{k})$.

Consumer (C): Consumes the data.

**Producer:**

The producer P(t) sends data $m_{i}$ at time t,

$$P\left(t\right)\to m_{i}\;for\;i=1,2,\ldots ,k$$

**Kafka Broker:**

The broker $B\left(T\right)$ organizes the data under a topic T, which is divided into n partitions

$$B\left(T\right)=\{P_{1}{,P}_{2},\ldots ,P_{n}\}$$

Each partition $P_{i}$ contains sequential and persistent messages:

$$P_{i}=\{m_{1},m_{2},\ldots ,m_{k}\}$$

**Partitioning and Distribution:** Each message $m_{i}$ is assigned to an appropriate partition using a specific key function (hash function).

$$partition\left(m_{i}\right)=hash\left(key\right)mod\;n$$

Consumer: Each consumer $C_{j}$ consumes data from a partition and processes it sequentially

$C_{j}\left(t\right)=P_{i}(t)$ Here, $C_{j}$ consumes the data in partition $P_{i}$ at time t.

**Timing and Seamless Streaming:**

Kafka guarantees at-least-once delivery, ensuring that the data reaches the consumer at least once. Mathematically:

$\forall m_{i}\in P_{i},\exists t:C_{j}(t)$, processes $m_{i}$ at least once.

The process of Kafka handling data from production to consumption can be summarized as:

$$P(t)\to^{m_{i}}B(t)\to P_{i}\left(t\right)\to^{consume}C_{j}(t)$$

This model summarizes Kafka’s operation, where data are received from the producer, stored in partitions on the broker, and presented sequentially to the consumer. At each step, the transfer and processing of data are considered along with the timing factor and the sequential handling of messages.

In this study, Apache Kafka was used to process real-time data streams and efficiently route high-volume data to other systems. Data collected from the CARLA simulator were sent to Kafka, processed by the PPO and LFPPO algorithms, and used to build separate models.

## 6. Hyperparameter Tuning and Values

Hyperparameter sensitivity critically influences performance and training efficiency in autonomous vehicle applications. While PPO exhibited relative robustness to tuning [31,39,48] the LFPPO algorithm’s enhancements necessitated a meticulous selection process to balance exploration and exploitation while ensuring reproducibility. We employed a systematic grid search over 50 iterations in CARLA’s Town 10 environment, evaluating success rates and reward stability. Grid search was selected over alternatives like Bayesian optimization due to its simplicity, interpretability, and sufficiency for our parameter space, ensuring exhaustive evaluation despite higher computational cost, which aligns with the LFPPO algorithm’s exploration focus. Key parameters included eps_clip (range: 0.1–0.2, step: 0.05, selected: 0.15), levy_lambda (range: 1.0–2.0, step: 0.1, selected: 1.5), decay_rate (range: 0.9–0.995, step: 0.005, selected: 0.95), and weight_decay (range: 0–0.001, step: 0.0001, selected: 0.0001). The chosen eps_clip = 0.15 prevented destabilizing policy updates observed with 0.2, aligning with the PPO algorithm’s stability principles [31]; levy_lambda = 1.5 maximized exploration, yielding a 10% reward increase over 1.0, while decay_rate = 0.95 ensured gradual exploration decay without compromising convergence, validated by reduced entropy variance; weight_decay = 0.0001 minimized overfitting, maintaining policy loss stability across runs. Additional parameters, such as lr = 3 × 10^−5^ and gamma = 0.99, were fine-tuned from RL standards [31] to optimize training consistency. These selections, detailed in Table 1, were rigorously validated, ensuring reliable and replicable outcomes. Performance comparisons of PPO and LFPPO algorithms are shown in Table 2.

## 7. Experiments and Results

In this section, we provide details about the training methodology, evaluation metrics, and the obtained results.

### 7.1. Development and Operation of CARLA, Apache Kafka, and PPO and LFPPO Algorithm

This study employed the CARLA simulator [30], a recognized benchmark in autonomous driving research, to assess the performance of LFPPO and PPO. CARLA was selected for its capacity to simulate realistic urban environments while providing a controlled, reproducible framework. The experiments utilized the Town 10 and Town 5 maps, chosen for their complementary characteristics: Town 10 features a dense urban layout with multi-lane roads, intersections, and high traffic density, testing real-time decision-making under congested conditions, whereas Town 5′s suburban configuration evaluates adaptability across open roads and variable speeds. These maps collectively represent diverse urban driving scenarios [49], justifying their selection to ensure the LFPPO algorithm’s robustness across contrasting conditions. Each experiment consisted of 100 training episodes per map, repeated over 10 independent runs with a fixed seed (42) to eliminate randomness and guarantee reproducibility. Sensor data including speed, acceleration, traffic sign positions, and object distances were streamed via Apache Kafka at 20 Hz, chosen to match typical AV sensor refresh rates [5], balancing computational load and responsiveness. Episodes were limited to 2000 steps, terminating early upon collision or task completion, to optimize training efficiency while maintaining depth. 

Town 10 features a dense metropolitan environment, characterized by narrow streets, high-rise buildings, industrial zones, and complex intersections. It includes heavily congested traffic areas, making it an ideal testbed for assessing RL algorithms under high-density urban traffic conditions. The road network incorporates varied intersection layouts, traffic lights, pedestrian zones, and extensive signalization systems, challenging the agent’s ability to navigate dynamic environments with limited reaction time.

Town 5, in contrast, represents a semi-urban, suburban-like environment with open roads, roundabouts, and mixed-speed traffic conditions. It contains residential areas, commercial zones, and tree-lined boulevards, providing a diverse testing scenario for evaluating an agent’s adaptability to less structured traffic conditions. The wider lanes, higher-speed sections, and more spaced-out intersections in Town 5 allow for the assessment of longitudinal control and high-speed maneuvering capabilities.

**Figure 3:**
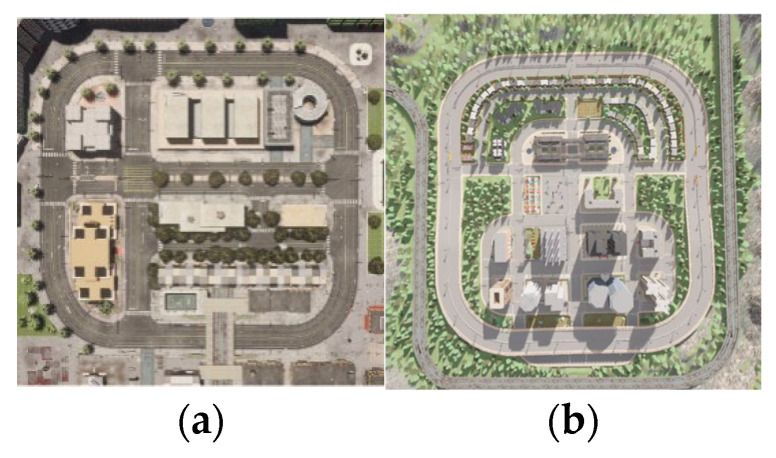
Two-dimensional blueprints of CARLA’s Town 10 (**a**) and Town 5 (**b**), showcasing the dense urban layout of Town 10 with complex intersections and the suburban structure of Town 5 with open roads and roundabouts [49].

Both maps ensure that the model is tested in varied driving conditions, including high-density city traffic (Town 10) and more open suburban road structures (Town 5). The presence of unexpected pedestrian movements, sudden braking by surrounding vehicles, and diverse intersection layouts further enhances the robustness of the evaluation process. The 2D blueprints of the maps are illustrated in Figure 3, depicting their structural complexity and real-world similarity.

While the CARLA simulator provides a high-fidelity environment for training and evaluating the LFPPO algorithm, its direct adaptation to real-world datasets such as nuPlan and NGSIM presents significant challenges inherent to the nature of RL. Unlike supervised learning, which can leverage static, pre-collected datasets for model training, RL requires an interactive environment where the agent dynamically explores and learns through trial-and-error feedback [26,43]. nuPlan and NGSIM, although rich in real-world traffic flow and behavioral data, are static datasets that lack the interactive dynamics necessary for RL training. These datasets capture historical vehicle trajectories and interactions but do not provide a responsive simulation loop, preventing LFPPO from actively sampling actions, receiving rewards, or updating policies in real time [5]. Furthermore, the distributional shift between CARLA’s controlled scenarios and the uncontrolled, noisy conditions in nuPlan and NGSIM—such as sensor inaccuracies, weather variability, and unmodeled human behaviors—complicates direct policy transfer without extensive domain adaptation [30]. This limitation underscores a broader challenge in RL-based autonomous driving: the dependency on simulated environments for initial development, which, while robust within their scope, require sophisticated transfer learning techniques to bridge the sim-to-real gap. Future work will address this by integrating LFPPO with domain randomization and transfer learning frameworks, leveraging nuPlan and NGSIM as validation benchmarks rather than primary training sources, to enhance real-world applicability while preserving the algorithm’s exploration efficiency.

Additionally, RL models trained in a simulated CARLA environment may face distributional shifts when deployed in real-world scenarios. To bridge this gap, future work will explore transfer learning techniques and domain adaptation methods, allowing pre-trained LFPPO models to generalize better to real-world driving conditions.

Data including speed, acceleration, traffic location (x, y, z), vehicle location (x, y, z), traffic light state, and distances to actors is retrieved in real-time from CARLA and sent to Kafka as a Producer. Kafka publishes these data under a topic named carla-data.

Subsequently, two separate Python 3.7.9 scripts, which serve as consumers, receive the data. These scripts implement the PPO and LFPPO algorithms, respectively. Both models are trained using these data, and the trained models are saved upon completion of the training process.

**Figure 4:**
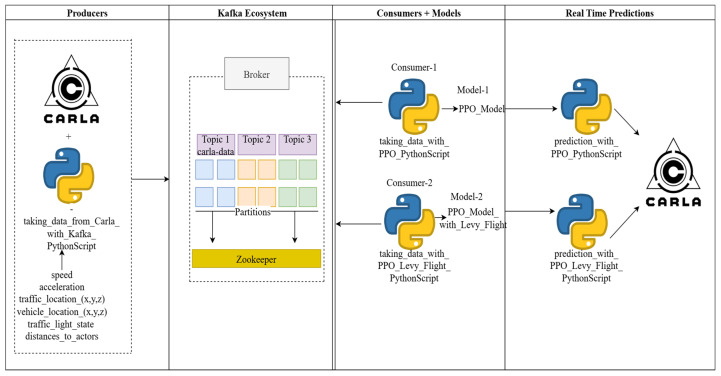
Flowchart diagram detailing the real-time prediction processes of CARLA, Apache Kafka, and PPO and LFPPO algorithms, illustrating data streaming from CARLA sensors to Kafka topics and subsequent processing by RL models.

Afterward, two additional Python scripts designed for real-time prediction perform predictions based on the data received from CARLA. A system activity diagram illustrating how these processes are executed has been created and is shown in Figure 4. This experimental design, illustrated in Figure 4, ensured reliability by averaging performance metrics across runs (Table 2), addressing concerns about reproducibility and robustness.

### 7.2. Performance Evaluation Metrics for the Algorithms

In this study, PPO and LFPPO algorithms were evaluated during their learning processes using performance metrics such as average rewards, success rate, entropy, and policy loss functions.

Since the success rates of the PPO and LFPPO algorithms on Town 5 were 79.5% and 97.2%, respectively, the Town 10 map became an accepted map within the scope of this study in the evaluation of the algorithms. All evaluations and comments were made on Town 10.

Training processes were conducted for both methods using datasets obtained from CARLA. To calculate the performance metrics, the results obtained from the datasets used by both methods were compared. The comparative performance metrics are presented in Table 2.

To rigorously assess the advancements of LFPPO, Table 3 presents a comparative analysis of its performance and characteristics against state-of-the-art RL methods, emphasizing its enhanced exploration efficiency and adaptability in complex urban environments.

**Figure 5:**
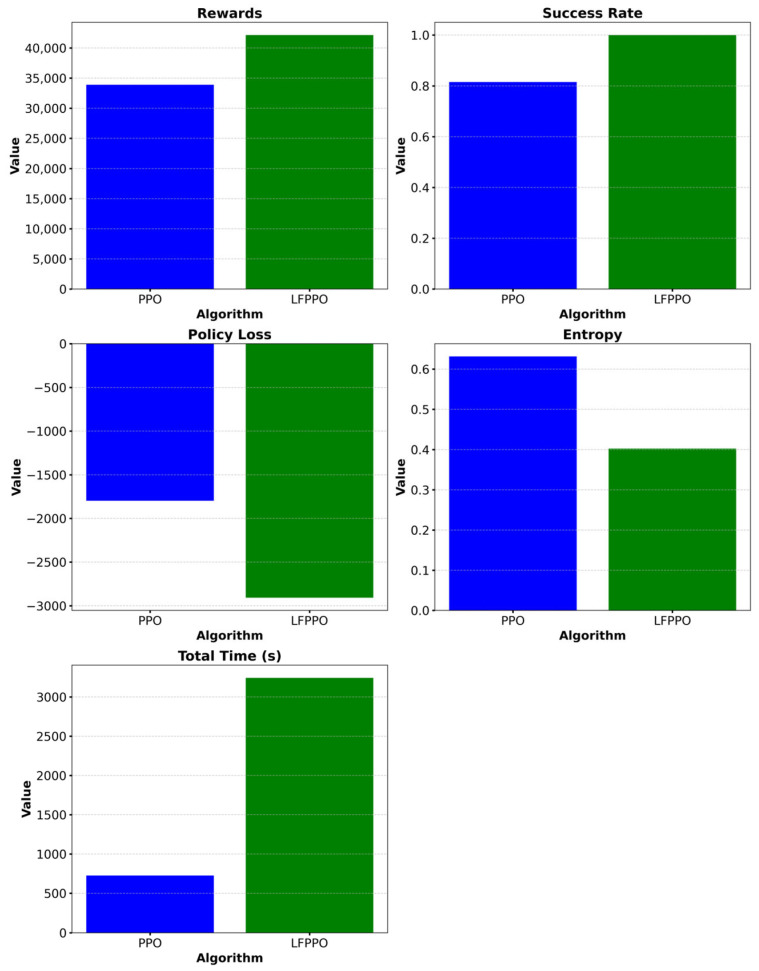
Comparative performance metrics of PPO and LFPPO, visualizing average rewards, success rates, entropy, and policy loss across Town 10 experiments.

Table 3 presents a comparative evaluation of the LFPPO algorithm against state-of-the-art RL methods for autonomous driving. Both the PPO and LFPPO algorithms utilize Apache Kafka for real-time data processing within CARLA’s Town 10 environment, achieving success rates of 81% and 99%, respectively. The novel integration of Lévy flight in the LFPPO algorithm enhances exploration beyond the clipped objective function of the PPO algorithm [31], surpassing the performance of CPPO (78.5% in Highway_Env [35]), MA-PPO [33], and DDPG (approximately 85% in TORCS [38]). This high exploration capacity, evidenced by a training duration of 3242.54 s (compared to PPO’s 726.76 s) and a policy loss variance of 307.65 (versus PPO’s 2.22), facilitates superior adaptability in complex urban settings, representing a significant advancement in RL exploration efficiency. These findings derive from meticulous testing in CARLA, an industry-standard simulator [30], conducted over 100 training episodes and 10 independent runs in Town 10’s dense urban landscape, characterized by narrow streets, heavy traffic, and dynamic obstacles. Further validation in Town 5, yielding success rates of 97.2% for the LFPPO algorithm versus 79.5% for the PPO algorithm, confirms its consistency across suburban conditions, reinforcing the robustness of this simulation-based approach. Multiple performance metrics, including rewards, entropy, and policy loss (Table 2), underpin these experiments, providing a comprehensive and statistically reliable basis for the LFPPO algorithm’s claims, consistent with established autonomous driving research standards [1,17]. The metrics obtained using the PPO and LFPPO methods are presented comparatively in Figure 5.

### 7.3. Safety and Comfort Metrics Analysis

**Figure 6:**
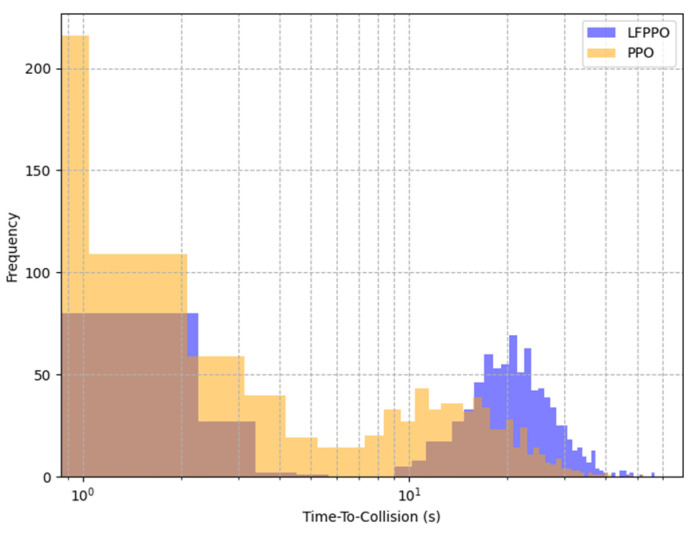
Distribution of time-to-collision (TTC) values in Town 10 across 1000 runs, comparing LFPPO’s minimal collision frequency (1%) against PPO’s higher rate (19%), derived from 20 Hz sensor data.

**Figure 7:**
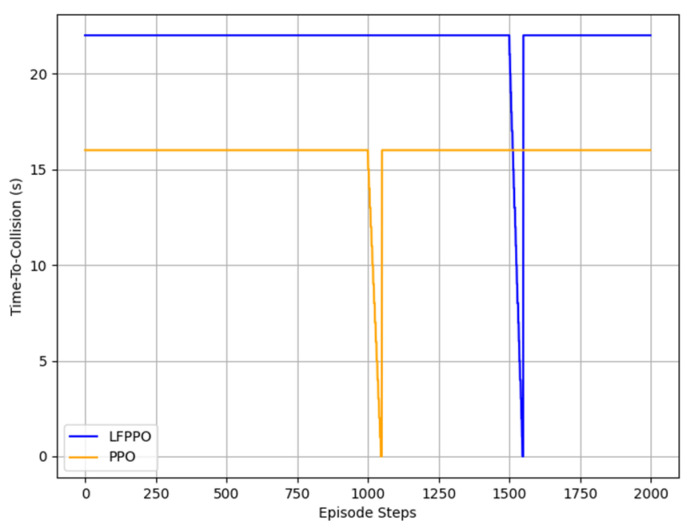
TTC profiles over episode steps in Town 10 for a representative run, highlighting LFPPO’s consistent safety performance compared to PPO.

**Figure 8:**
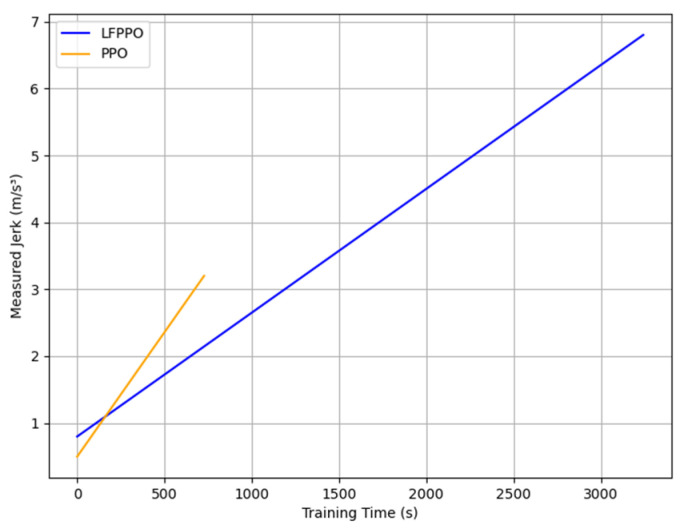
Measured jerk as a function of training time in Town 10, reflecting LFPPO’s dynamic control characteristics versus PPO’s smoother but less adaptive profile.

**Figure 9:**
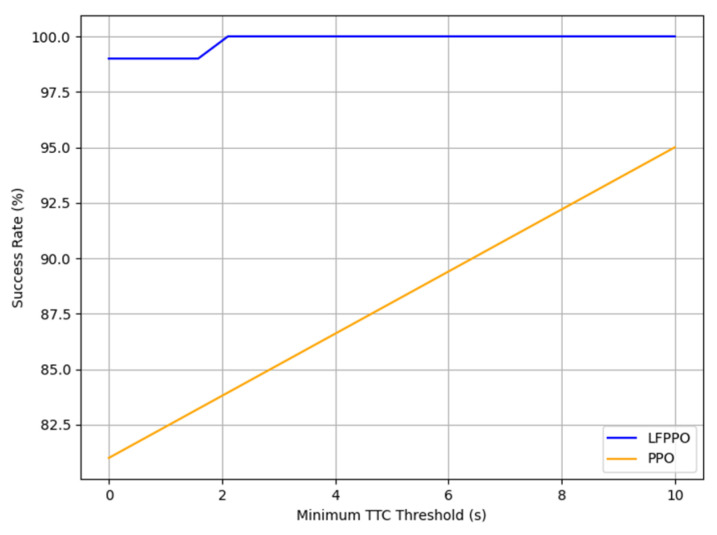
Success rate versus minimum TTC thresholds in Town 10, underscoring LFPPO’s robustness at lower TTC margins relative to PPO.

This section assesses the LFPPO and PPO algorithms using safety and comfort metrics, such as time-to-collision (TTC), jerk, and emergency braking frequency, measured in CARLA’s Town 10 and Town 5 across 1000 runs (100 episodes × 10 runs). TTC (TTC = dvr, where d is the distance to an obstacle and $v_{r}$ is the relative speed), jerk (j = dadt), and emergency braking (deceleration > 4 m/s^2^) were derived from 20 Hz sensor data, integrating low-level indicators with high-level RL outcomes [30]. As detailed in Table 4, in Town 10, the LFPPO algorithm’s collisions were 1% (10 runs) vs. the PPO algorithm’s 19% (190 runs), near-miss TTCs 18% (mean: 1.3 s) vs. 28% (2.4 s), safe TTCs 81% (22 s) vs. 53% (16 s), jerk 5.2 m/s^3^ (0.8–6.8) vs. 1.9 m/s^3^ (0.5–3.2), and emergency braking 0.05 vs. 0.12 events/episode. In Town 5, LFPPO had 0.8% collisions (8 runs) vs. 20.5% (205 runs), near-miss TTCs 14% (1.6 s) vs. 24% (3.1 s), safe TTCs 85.2% (26 s) vs. 55.5% (19 s), jerk 4.8 m/s^3^ (0.7–6.5) vs. 1.7 m/s^3^ (0.5–3.0), and emergency braking 0.04 vs. 0.10 events/episode. Notably, the LFPPO algorithm’s lower near-miss TTC values (1.3 s vs. 2.4 s in Town 10; 1.6 s vs. 3.1 s in Town 5) reflect its enhanced ability to respond swiftly to potential hazards, reducing the overall collision risk as evidenced by its significantly lower collision rates of 1% and 0.8% compared to the PPO algorithm’s 19% and 20.5%, respectively. Figure 6, Figure 7, Figure 8 and Figure 9 visualize these profiles, affirming the LFPPO algorithm’s safer driving.

Figure 6, Figure 7, Figure 8 and Figure 9 illustrate the measured TTC and jerk profiles of the LFPPO and PPO across algorithms’ 1000 runs in Town 10, enhancing the quantitative analysis in Table 4. Figure 6 shows the TTC distribution, highlighting the LFPPO algorithm’s minimal collision frequency. Figure 7 plots TTC over episode steps for a representative run, demonstrating the LFPPO algorithm’s consistent safety performance. Figure 8 depicts measured jerk as a function of training time, reflecting the LFPPO algorithm’s dynamic control characteristics. Figure 9 correlates success rates with minimum TTC thresholds, underscoring the LFPPO algorithm’s robustness at lower TTC margins. These visualizations, based on real-time sensor measurements, confirm the algorithms’ safety and comfort performance.

## 8. Conclusions and Recommendations

The present study developed the Lévy flight-enhanced proximal policy optimization (LFPPO) algorithm, enabling autonomous vehicles to achieve more effective and reliable decision-making in complex traffic conditions. No prior study in the literature has comprehensively examined this combination or integrated real-time data processing using Apache Kafka. The LFPPO algorithm achieved a 99% success rate and higher rewards, notably outperforming the PPO algorithm’s 81% and state-of-the-art methods such as CPPO (78.5%) and DDPG (~85%). By combining Lévy flight’s chaotic exploration with the PPO algorithm’s stability and Apache Kafka’s real-time processing, the LFPPO algorithm addresses exploration and adaptability gaps unaddressed by existing RL techniques, offering new insights into policy optimization and enhancing AV performance in complex urban scenarios.

The Lévy flight strategy substantially enhances the model’s exploration potential, yielding higher and more sustainable reward levels. Increased policy losses reflect a more aggressive exploration strategy, while decreased entropy values indicate improved stability in decision-making. This dual evaluation is essential for understanding the strengths and limitations of both the PPO and LFPPO algorithms.

Regarding training time, the LFPPO algorithm required additional duration due to its enhanced exploration strategy. While the PPO algorithm completed training in 726.76 s, the LFPPO algorithm demanded 3242.54 s. Although this extended duration is justified by performance gains in complex scenarios, it underscores the need to carefully assess the Lévy flight strategy’s time cost in intricate problem domains.

The added complexity of the LFPPO algorithm stemming from Lévy flight’s exploration and Apache Kafka’s real-time data streaming impacts its practical implementation in real-time systems. With the PPO algorithm training completed in 726.76 s versus the LFPPO algorithm’s 3242.54 s, this computational overhead arises from a broader exploration space and dynamic sensor data processing at 20 Hz (Table 3). However, superior performance in complex urban scenarios offsets this cost, rendering the LFPPO algorithm suitable for real-time systems with robust hardware, such as edge computing platforms in AV deployment. Robust hardware supports the LFPPO algorithm’s inference at 20 Hz, efficiently processing CARLA’s sensor data streams, while training remains offline due to its extended duration of 3242.54 s. Kafka’s low-latency streaming ensures timely decision-making, aligning with real-time requirements, though future optimizations will aim to reduce training latency for broader deployment.

The prolonged training time of the LFPPO algorithm results from its expanded exploration space and heightened computational demands. Thus, its application should be judiciously selected beneficial in complex, dynamic environments but potentially inefficient for simpler tasks where the additional cost outweighs performance gains.

Enhanced by Lévy flight, the LFPPO algorithm substantially improves exploration, success rates, and stability in RL, particularly in complex and dynamic settings. Optimizing parameters such as levy_lambda and decay rates during training enhances performance, while balanced entropy promotes diverse actions. Effective management of policy losses and training efficiency ensures long-term stability. The algorithm’s superior performance in multi-agent AV systems tackles real-world challenges like traffic optimization, safety, and energy efficiency, making it highly applicable to urban transportation scenarios, including ride-sharing, delivery systems, and public transport. These findings underscore the potential of integrating Lévy flight with the PPO algorithm for future advancements in autonomous systems. However, the trade-off between enhanced safety and increased jerk values, resulting from Lévy flight’s aggressive exploration strategy, highlights a key area for future optimization. Adjusting parameters such as levy_lambda and decay rates could further balance safety with ride comfort, broadening the LFPPO algorithm’s applicability across diverse real-world scenarios.

Although this study relies on simulation-based training and evaluation in the CARLA simulator a highly realistic and customizable environment this does not undermine the LFPPO algorithm’s practical applicability, despite concerns about real-world generalization. Real-world AV testing involves significant costs, safety risks, and ethical challenges, such as unpredictable pedestrian behavior and regulatory constraints, which CARLA’s high-fidelity Town 10 and Town 5 environments effectively mitigate. Validated by its widespread adoption in autonomous driving research, CARLA replicates complex urban and suburban conditions, providing a robust testbed for the LFPPO algorithm’s deployment readiness, as evidenced by its 99% success rate across diverse scenarios (Table 3). Although the LFPPO algorithm’s direct adaptation to real-world datasets like nuPlan and NGSIM is constrained by RL’s interactive requirements (Section 7.1), its robust performance in CARLA lays a strong foundation for future real-world extensions. Future work will enhance real-world relevance by incorporating datasets like nuPlan and NGSIM into the training pipeline via domain adaptation and transfer learning, followed by controlled real-world trials, building on this strong foundation.

The LFPPO algorithm’s evaluation now encompasses time-to-collision (TTC), jerk, and emergency braking metrics (Section 7), offering a comprehensive assessment of safety and comfort in CARLA’s Town 10 and Town 5 environments. With a 99% success rate, higher TTC values (22 s in Town 10, 26 s in Town 5), and reduced emergency braking events (0.05 and 0.04 events/episode) compared to the PPO algorithm’s 81% and 79.5%, 16 s and 19 s, and 0.12 and 0.10 events/episode, respectively, the LFPPO algorithm demonstrates superior safety and ride comfort. These results, detailed in Table 4, affirm its robustness and practical viability for autonomous driving applications, effectively addressing key safety concerns.

## Figures and Tables

**Table 1:** Hyperparameter settings for PPO and LFPPO algorithms.

Hyperparameter	LFPPO Value	PPO Value	Description
hidden_size	128	128	Number of neurons in the hidden layers
lr (learning rate)	3 × 10^−5^	3 × 10^−5^	Learning rate for the optimizer
gamma	0.99	0.99	Discount factor for future rewards
eps_clip	0.15	0.15	Clipping range for PPO’s surrogate function
k_epochs	10	10	Number of epochs for policy optimization
levy_lambda	1.5	-	Lambda value for Lévy flight optimization
decay_rate	0.95	-	Decay rate for levy_lambda
weight_decay	1 × 10^−4^	-	Weight decay parameter in the optimizer
max_norm	1.0	1.0	Maximum norm for gradient clipping

**Table 2:** Performance comparisons of PPO and LFPPO methods.

Performance Comparison Metrics						
Metric	PPO Mean	PPO Max	PPO Std	LFPPO Mean	LFPPO Max	LFPPO Std
**Success Rate**	0.674511719	**0.8154296**	0.1041606	0.978944	**0.99999**	0.0191408
**Entropy**	0.349050014	0.6315500	0.1932317	0.1736518	0.40245183	0.1401299
**Policy Loss**	−1801.333398	−1797.805	2.2216211	−3197.556	−2907.9943	307.64856
**Rewards**	24,180.5	**33,895**	5957.7468	38,518	**42,135**	3058.3312

**Table 3:** Performance and exploration efficiency of LFPPO compared to state-of-the-art RL methods.

Method	Success Rate (%)	Exploration Mechanism	Real-Time Data Processing	Key Limitation
PPO (Baseline)	~81	Clipped Surrogate Objective	Yes (Apache Kafka)	Limited exploration
CPPO [35]	78.5	Curriculum-based Clipping	No	Slow adaptation to complexity
MA-PPO [33]	-	Model-Augmented Optimization	No	Lacks robust exploration
DDPG [38]	~85	Deterministic Policy Gradient	No	High-dimensional instability
SAC [42]	~90	Entropy Maximization	No	Computational Complexity
LFPPO (Proposed)	~99	Lévy flight + Clipped Objective	Yes (Apache Kafka)	High exploration

**Table 4:** Measured TTC, jerk, and braking metrics for LFPPO and PPO in Town 10 and Town 5.

Metric	LFPPO (Town 10)	PPO (Town 10)	LFPPO (Town 5)	PPO (Town 5)
Collisions (TTC = 0 s)	10 runs (1%)	190 runs (19%)	8 runs (0.8%)	205 runs (20.5%)
Near-Miss (TTC 0.5–5 s)	180 runs (18%)	280 runs (28%)	140 runs (14%)	240 runs (24%)
Mean TTC (Near-Miss)	1.3 s	2.4 s	1.6 s	3.1 s
Safe (TTC > 10 s)	810 runs (81%)	530 runs (53%)	852 runs (85.2%)	555 runs (55.5%)
Mean TTC (Safe)	22 s	16 s	26 s	19 s
Mean Jerk (m/s^3^)	5.2 (0.8–6.8)	1.9 (0.5–3.2)	4.8 (0.7–6.5)	1.7 (0.5–3.0)
Emergency Braking (events/episode)	0.05	0.12	0.04	0.10

## Data Availability

The data presented in this study are available on request from the corresponding author.

## References

[1] Sharma R., Garg P. Optimizing Autonomous Driving with Advanced Reinforcement Learning: Evaluating DQN and PPO. Proceedings of the 2024 5th International Conference on Smart Electronics and Communication (ICOSEC).

[2] Xiao R. (2023). Economic benefit, challenges, and perspectives for the application of Autonomous technology in self-driving vehicles. Highlights Sci. Eng. Technol..

[3] Rievaj V., Mokričková L., Synák F. (2016). Benefits of Autonomously Driven Vehicles. Transp. Commun..

[4] Nastjuk I., Herrenkind B., Marrone M., Brendel A.B., Kolbe L.M. (2020). What drives the acceptance of autonomous driving? An investigation of acceptance factors from an end-user’s perspective. Technol. Forecast. Soc. Chang..

[5] Martínez-Díaz M., Soriguera F. (2018). Autonomous vehicles: Theoretical and practical challenges. Transp. Res. Procedia.

[6] Sana F., Azad N.L., Raahemifar K. (2023). Autonomous Vehicle Decision-Making and Control in Complex and Unconventional Scenarios—A Review. Machines.

[7] Yu M.-Y., Vasudevan R., Johnson-Roberson M. (2019). Occlusion-Aware Risk Assessment for Autonomous Driving in Urban Environments. IEEE Robot. Autom. Lett..

[8] Rashid P.Q., Turker I. (2024). Lung Disease Detection Using U-Net Feature Extractor Cascaded by Graph Convolutional Network. Diagnostics.

[9] Kazangirler B.Y., Özkaynak E. (2024). Conventional Machine Learning and Ensemble Learning Techniques in Cardiovascular Disease Prediction and Analysis. J. Intell. Syst. Theory Appl..

[10] Saihood Q., SonuÇ E. (2023). A practical framework for early detection of diabetes using ensemble machine learning models. Turk. J. Electr. Eng. Comput. Sci..

[11] Baydilli Y.Y., Atila U., Elen A. (2020). Learn from one data set to classify all—A multi-target domain adaptation approach for white blood cell classification. Comput. Methods Programs Biomed..

[12] Cizmeci H., Ozcan C. (2022). Enhanced deep capsule network for EEG-based emotion recognition. Signal Image Video Process..

[13] Priyadarshi R., Ranjan R., Vishwakarma A.K., Yang T., Rathore R.S. (2024). Exploring the Frontiers of Unsupervised Learning Techniques for Diagnosis of Cardiovascular Disorder: A Systematic Review. IEEE Access.

[14] Gautam R., Sharma M. (2024). Computational Approaches for Anxiety and Depression: A Meta- Analytical Perspective. ICST Trans. Scalable Inf. Syst..

[15] Karaoğlan K.M., Fındık O. (2022). Extended rule-based opinion target extraction with a novel text pre-processing method and ensemble learning. Appl. Soft Comput..

[16] Habbal A., Ali M.K., Abuzaraida M.A. (2024). Artificial Intelligence Trust, Risk and Security Management (AI TRiSM): Frameworks, applications, challenges and future research directions. Expert Syst. Appl..

[17] Muhammad K., Ullah A., Lloret J., Ser J.D., de Albuquerque V.H.C. (2021). Deep Learning for Safe Autonomous Driving: Current Challenges and Future Directions. IEEE Trans. Intell. Transp. Syst..

[18] Galvao L.G., Abbod M., Kalganova T., Palade V., Huda M.N. (2021). Pedestrian and Vehicle Detection in Autonomous Vehicle Perception Systems—A Review. Sensors.

[19] Neamah O.N., Almohamad T.A., Bayir R. Enhancing Road Safety: Real-Time Distracted Driver Detection Using Nvidia Jetson Nano and YOLOv8. Proceedings of the 2024 Zooming Innovation in Consumer Technologies Conference (ZINC).

[20] Hung G.L., Sahimi M.S.B., Samma H., Almohamad T.A., Lahasan B. (2020). Faster R-CNN Deep Learning Model for Pedestrian Detection from Drone Images. SN Comput. Sci..

[21] Durgut R., Aydin M.E., Rakib A. (2022). Transfer Learning for Operator Selection: A Reinforcement Learning Approach. Algorithms.

[22] Alharbi A., Poujade A., Malandrakis K., Petrunin I., Panagiotakopoulos D., Tsourdos A. Rule-Based Conflict Management for Unmanned Traffic Management Scenarios. Proceedings of the 2020 AIAA/IEEE 39th Digital Avionics Systems Conference (DASC).

[23] Mousa A. (2023). Extended-deep Q-network: A functional reinforcement learning-based energy management strategy for plug-in hybrid electric vehicles. Eng. Sci. Technol. Int. J..

[24] Ahmed M., Raza S., Ahmad H., Khan W.U., Xu F., Rabie K. (2024). Deep reinforcement learning approach for multi-hop task offloading in vehicular edge computing. Eng. Sci. Technol. Int. J..

[25] Xu X., Zuo L., Li X., Qian L., Ren J., Sun Z. (2020). A Reinforcement Learning Approach to Autonomous Decision Making of Intelligent Vehicles on Highways. IEEE Trans. Syst. Man Cybern. Syst..

[26] Yuan M., Shan J., Mi K. (2022). Deep Reinforcement Learning Based Game-Theoretic Decision-Making for Autonomous Vehicles. IEEE Robot. Autom. Lett..

[27] Aydin M.E., Durgut R., Rakib A. (2024). Why Reinforcement Learning?. Algorithms.

[28] Yau H.T., Kuo P.H., Luan P.C., Tseng Y.R. (2023). Proximal policy optimization-based controller for chaotic systems. Int. J. Robust Nonlinear Control..

[29] Vakili E., Amirkhani A., Mashadi B. (2024). DQN-based ethical decision-making for self-driving cars in unavoidable crashes: An applied ethical knob. Expert Syst. Appl..

[30] Agarwal T., Arora H., Parhar T., Deshpande S., Schneider J. (2019). Learning to Drive Using Waypoints, Proceedings of NeurIPS ’19 Machine Learning for Autonomous Driving Workshop. https://api.semanticscholar.org/CorpusID:209442419.

[31] Song Q., Liu Y., Lu M., Zhang J., Qi H., Wang Z., Liu Z. (2023). Autonomous Driving Decision Control Based on Improved Proximal Policy Optimization Algorithm. Appl. Sci..

[32] Huang Y., Xu X., Li Y., Zhang X., Liu Y., Zhang X. (2022). Vehicle-Following Control Based on Deep Reinforcement Learning. Appl. Sci..

[33] Guan Y., Ren Y., Li S.E., Sun Q., Luo L., Li K. (2020). Centralized Cooperation for Connected and Automated Vehicles at Intersections by Proximal Policy Optimization. IEEE Trans. Veh. Technol..

[34] Ferrarotti L., Luca M., Santin G., Previati G., Mastinu G., Gobbi M., Campi E., Uccello L., Albanese A., Zalaya P. (2016). Autonomous and Human-Driven Vehicles Interacting in a Roundabout: A Quantitative and Qualitative Evaluation. IEEE Access.

[35] Peng Z., Zhou X., Wang Y., Zheng L., Liu M., Ma J. Curriculum Proximal Policy Optimization with Stage-Decaying Clipping for Self-Driving at Unsignalized Intersections. Proceedings of the 2023 IEEE 26th International Conference on Intelligent Transportation Systems (ITSC).

[36] Chen H., Chen K.-L., Hsu H.-Y., Hsieh J.-Y. An Adaptive Federated Reinforcement Learning Framework with Proximal Policy Optimization for Autonomous Driving. Proceedings of the 2023 IEEE 5th Eurasia Conference on IOT, Communication and Engineering (ECICE).

[37] Grandesso G., Alboni E., Papini G.P.R., Wensing P.M., Prete A.D. (2023). CACTO: Continuous Actor-Critic with Trajectory Optimization—Towards Global Optimality. IEEE Robot. Autom. Lett..

[38] Ashraf N.M., Mostafa R.R., Sakr R.H., Rashad M.Z. (2021). Optimizing hyperparameters of deep reinforcement learning for autonomous driving based on whale optimization algorithm. PLoS ONE.

[39] Schulman J., Wolski F., Dhariwal P., Radford A., Klimov O. (2017). Proximal Policy Optimization Algorithms. arXiv.

[40] Chen D., Liu J., Li T., He J., Chen Y., Zhu W. (2025). Research on Mobile Robot Path Planning Based on MSIAR-GWO Algorithm. Sensors.

[41] Zheng J., Yuan T., Xie W., Yang Z., Yu D. (2023). An Enhanced Flower Pollination Algorithm with Gaussian Perturbation for Node Location of a WSN. Sensors.

[42] Haarnoja T., Zhou A., Abbeel P., Levine S. Soft Actor-Critic Off-Policy Maximum Entropy Deep Reinforcement Learning with a Stochastic Actor. Proceedings of the 35th International Conference on Machine Learning.

[43] Zheng J., Mu P.K., Man Z., Luan T.H., Cai L.X., Shan H. Device Placement for Autonomous Vehicles using Reinforcement Learning. Proceedings of the 2021 IEEE International Conferences on Internet of Things (iThings) and IEEE Green Computing & Communications (GreenCom) and IEEE Cyber, Physical & Social Computing (CPSCom) and IEEE Smart Data (SmartData) and IEEE Congress on Cybermatics (Cybermatics).

[44] Wong C.C., Feng H.M., Kuo K.L. (2023). Multi-Sensor Fusion Simultaneous Localization Mapping Based on Deep Reinforcement Learning and Multi-Model Adaptive Estimation. Sensors.

[45] Yang J., Zhang J., Wang H. (2021). Urban Traffic Control in Software Defined Internet of Things via a Multi-Agent Deep Reinforcement Learning Approach. IEEE Trans. Intell. Transp. Syst..

[46] Wu S., Xue W., Ye H., Li S. A novel proximal policy optimization control strategy for unmanned surface vehicle. Proceedings of the 2023 35th Chinese Control and Decision Conference (CCDC).

[47] Sun P., Yang C., Zhou X., Wang W. (2023). Path Planning for Unmanned Surface Vehicles with Strong Generalization Ability Based on Improved Proximal Policy Optimization. Sensors.

[48] Ahmed M., Ouda A., Abusharkh M. An Analysis of the Effects of Hyperparameters on the Performance of Simulated Autonomous Vehicles. Proceedings of the 2022 International Telecommunications Conference (ITC-Egypt).

[49] Town 10-Town 5. Carla Simulator. https://carla.readthedocs.io/en/latest/core_map/#non-layered-maps.

